# Uncommon Cardiac Metastasis in Common Carcinoma Colon

**DOI:** 10.7759/cureus.21703

**Published:** 2022-01-28

**Authors:** Shravanthi Woodayagiri, Swathy Moorthy, Basith Ahmed, Mohanapriya G

**Affiliations:** 1 Internal Medicine, Sri Ramachandra Institute of Higher Education and Research, Chennai, IND; 2 General Surgery, Sri Ramachandra Institute of Higher Education and Research, Chennai, IND

**Keywords:** folfirinox, atrial fibrillation, malignancy, cardiac metastasis, ca colon

## Abstract

Colon carcinoma (CA) is one of the most common cancers worldwide. Cardiac metastasis in CA is quite rare with only a few incidences. These tumors are usually clinically silent and are discovered on autopsy. We present a case of a 62-year-old woman, known diabetic, hypertensive, and hypothyroid patient, who presented with complaints of abdominal distention and obstipation with multiple episodes of vomiting undigested food particles for three days. She had been passing dark tarry stools infrequently for over a month. She complained of a progressive loss of appetite and 5 kg weight loss over a month. Her examination revealed pallor and irregular pulse with a rate of 94/min. She had a distended non-tender abdomen and absent bowel sounds. Contrast-enhanced computed tomography (CECT) abdomen showed circumferential thickening of the descending colon, causing acute stenosis with multiple liver metastases and enlarged pericolic lymph nodes. Serum carcinoembryonic antigen (CEA) was elevated, 55.45 ng/mL. She underwent an emergency exploratory laparotomy with transverse loop colostomy. Histopathology report showed moderately differentiated adenocarcinoma. ECG showed atrial fibrillation and two-dimensional echocardiogram showed right ventricular metastasis. High-resolution computed tomography (HRCT) thorax was done to confirm the diagnosis.

The common sites of metastases from colorectal cancer are the lymph nodes, liver, and lungs. When cardiac metastasis occurs, it often presents with features of heart failure. Our patient presented with atrial fibrillation. As the incidence of cardiac metastasis is quite rare, there is no standard established treatment. Certain chemotherapeutic drugs, such as 5-fluorouracil, oxaliplatin, irinotecan (FOLFIRINOX regimen), have been shown to improve cardiac metastases. Due to the extensive spread of primary cancer in our patient, she was planned for palliative chemotherapy; however, the patient expired before initiation of therapy.

## Introduction

Colon carcinoma (CA) is the third most common cancer around the globe [1]. The common sites for metastases from CA are the lymph nodes, liver, and lungs. Cardiac metastases in CA are rare, with an incidence of 1.4-7.2%, according to autopsy studies [2]. To our knowledge, only 31 cases have been currently reported [3]. In this article, we discuss a case of CA with cardiac metastasis presenting with abdominal distention, obstipation, vomiting, melena, pallor, and irregular pulse. Therefore, description and evaluation of CA-related cardiac metastasis cases are necessary to understand the impact of cardiac metastasis on patients’ life and develop appropriate diagnostic and treatment strategies.

## Case presentation

**Figure 1:**
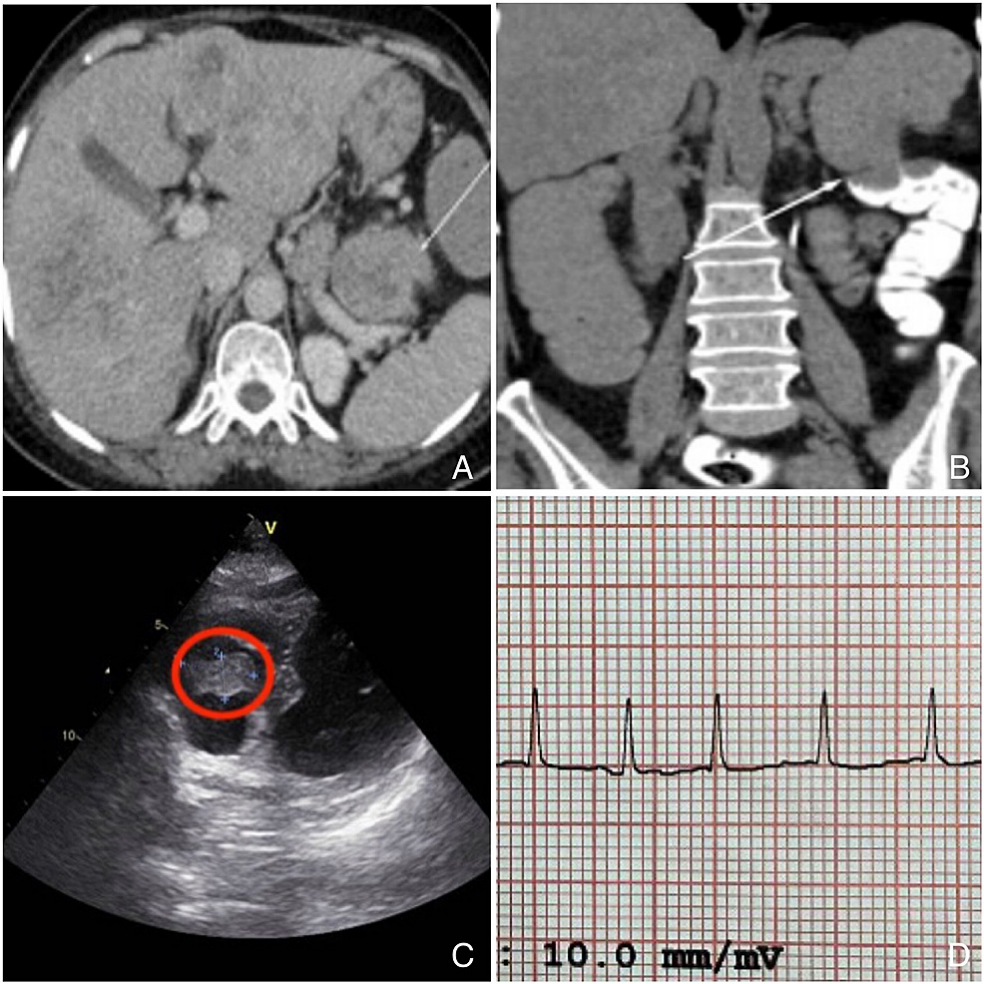
(A) Multiple liver metastases with regional lymphadenopathy, (B) circumferential thickening of proximal descending colon, (C) right atrial metastasis, and (D) atrial fibrillation.

We present a case of a 62-year-old woman with a history of diabetes, hypertension, and hypothyroidism. She complained of abdominal distention, obstipation, and multiple episodes of vomiting undigested food particles for three days. She had been passing dark tarry stools infrequently and complained of a progressive loss of appetite, fatigue, sleeplessness, and 5 kg weight loss over a month. No other significant complaints, history of addictions, or hospitalizations in the past were present. There was no family history of malignancy. Her examination revealed pallor and irregular pulse with a heart rate of 94 beats/min. She had a distended non-tender abdomen with full flanks, no organomegaly, and absent bowel sounds. Other system examinations revealed bilateral basal crepitations in the lungs with normal cardiac sounds. The rectal examination showed fecal staining and no palpable nodules. Baseline investigations revealed that hemoglobin (Hb) was 9.5 gm/dL, total white blood cell (WBC) count was 10,100 cells/mm^3^, platelets were 6.11 x 10^5 ^cells/mm^3^, international normalized ratio (INR) was 1.3, and HbA1c was 7.2%. Renal and liver functions were normal. Contrast-enhanced computed tomography (CECT) of the abdomen showed multiple liver metastases (the largest was 7.9 × 5.5 cm in the segment II of the left lobe) and enlarged pericolic lymph nodes (the largest was 14 × 9 mm) (Figure 1A). Malignant circumferential thickening (up to 2 cm) of the uppermost part of the descending colon for a length of about 5 cm, causing acute stenosis with upstream dilation of ileal loops was also seen (Figure 1B). Serum carcinoembryonic antigen (CEA) was elevated, 55.45 ng/mL. She underwent an emergency exploratory laparotomy with transverse loop colostomy. Peritoneal fluid was negative for malignant cells; however, the histopathology report showed moderately differentiated adenocarcinoma. Considering persistent hypertension (at admission blood pressure was 160/80 mmHg) and irregular rhythm, cardiac evaluation was done. A two-dimensional electrocardiogram (ECG) showed an irregularly shaped right ventricular mass of size 1.5 cm x 2.5 cm with normal chamber dimensions, no regional motion wall abnormality, and an ejection fraction of 62% (Figure 1C). And, an electrocardiogram showed atrial fibrillation (Figure 1D). Her cardiac enzymes were elevated: creatine phosphokinase (CPK) was 466 U/L, creatine kinase-myocardial band (CPK-MB) was 59 U/L, and B-type natriuretic peptide (BNP) was 556.6 pg/mL.

High-resolution computed tomography (HRCT) of the thorax showed a heterogenous filling defect in the right ventricle (1.5 × 2.4 × 1.2 cm) with an 8.5 mm enlarged right lower paratracheal node and minimal bilateral pleural effusion. Incidentally, a small hypodense nodule was found in the left thyroid lobe. The postoperative period was uneventful, and the patient recovered from the procedure without any incidents.

## Discussion

CA is the third most commonly encountered cancer worldwide [1]. The common sites of metastases for CA are the lymph nodes, liver, and lungs. The most commonly seen tumor type is adenocarcinoma, as was the case in our patient. The heart is rarely reported as a site of metastasis, and such metastases are often discovered many years after the diagnosis of primary cancer, most often on autopsies. The longest interval of such discovery reported is 17 years [4]. However, in our case, the presence of cardiac metastasis was detected along with the primary tumor. Studies have shown that the incidence of cardiac metastasis in patients with a malignancy might be underestimated because the cardiac lesions are clinically silent in most cases. A progressive metastatic mass of the heart may occasionally cause acute heart failure or superior vena cava syndrome, resulting in sudden death. When symptomatic, they usually present with features of heart failure, including dyspnea, palpitations, and edema. Our patient presented with atrial fibrillation without apparent features of cardiac failure. Atrial fibrillation has been documented to occur in secondary cardiac tumors infiltrating the myocardium [5].

Due to the rarity of diagnosis, most of the available data on cardiac metastasis has been from autopsy studies. Cardiac metastasis has been commonly associated with pleural mesothelioma, melanoma, and lung cancer. Various imaging modalities, including transthoracic/ transesophageal echocardiography, CT, or magnetic resonance imaging (MRI), have been shown effective in detecting cardiac metastasis. An overall interpretation of the MR imaging features was found to have a diagnostic accuracy of 0.92 (area under the curve) for determining a cardiac mass to be malignant (confirmed at histologic evaluation). Compared with CT, MR imaging offers higher temporal resolution and additional tissue characterization, and MR imaging does not expose patients to ionizing radiation. However, although access to cardiac MR imaging is increasing, it remains less available than echocardiography or CT [6].

Considering the rarity of cardiac metastasis, there is no standard treatment. Most reports have been from autopsy studies, as histopathological confirmation of cardiac metastasis is difficult to obtain. There have been reports showing the benefits of using chemotherapeutic drugs such as 5-fluorouracil, oxaliplatin, and irinotecan (FOLFIRINOX) in combination with vascular endothelial growth factor (VEGF) inhibitors, such as bevacizumab, in the treatment of cardiac tumors [7]. Surgical resection of the tumor has also been tried; Koizumi et al. reported that although surgery is rarely recommended for treating metastatic cardiac tumors, surgical treatment could be effective in obstructive and solitary lesions to provide symptom relief and increase life expectancy [8]. Among case reports review of 31 patients, 14 patients underwent only surgical resection of the cardiac tumor, three underwent only chemotherapy, four had surgery followed by chemotherapy, nine received no treatment, and two were lost to follow-up [3]. Due to the extensive spread of primary cancer in our patient, she was planned for palliative chemotherapy with a combination of capecitabine and oxaliplatin for six cycles, followed by maintenance with oxaliplatin. However, the patient died before the initiation of chemotherapy.

## Conclusions

We aim to highlight the importance of screening for presence of cardiac metastasis during the routine workup of colon cancer. They are often clinically silent and are detected after extensive metastasis have occurred. Recent studies have shown good response to FOLFIRINOX therapy in reducing the size of cardiac metastatic tumors. Early detection will help optimize treatment regimens and improve patient outcomes.
